# Chemical Constituents, Antimicrobial, Cytotoxicity, Mutagenic and Antimutagenic Effects of *Artemisia ciniformis*

**Published:** 2016

**Authors:** Mahboubeh Taherkhani

**Affiliations:** Department of Chemistry, College of Science, Takestan Branch, Islamic Azad University, Takestan, Iran

**Keywords:** *Artemisia ciniformis*, Antimicrobial, Cytotoxicity, Antimutagenic, HeLa and lymphocytes cells

## Abstract

The aim of this study was to determine the chemical constituents, antimicrobial, cytotoxicity, mutagenic and anti-mutagenic activities of the essential oil of *Artemisia ciniformis* Krasch. & Popov ex Poljakov, against important bacterial pathogens and human cells which were unknown before. *In-vitro* cytotoxicity was measured using a modified MTT assay on normal human lymphocytes and tumor HeLa cells. The mutagenic and antimutagenic activities of the oil were evaluated using the *Salmonella* *typhimurium* tester strains TA98 and TA100, together with nitrofluorene for TA98 and sodium azide for TA100 without (**-**S9) metabolic activation, and 2-aminoantracene for TA98 and TA100 with metabolic (+S9) activation. Oxygenated monoterpenes especially camphor (30.21%), 1,8-cineole (23.7%) and *trans*-Pinocarveol (12.28%) were the major components of the oil of *A. ciniformis. *Bactericidal kinetics of this oil indicated that *Acinetobacter baumannii* is the most vulnerable one (MIC = 0.02 mg/mL, MBC = 0.04 mg/mL, D_value_ = 3.57 min). The oil displayed an excellent cytotoxic action toward the human tumor cell line (IC_50 _= 19.64 µg/mL). The oil of *A. ciniformis *showed excellent antimutagenicity effect on the 2-nitrofluorene, in the strain of* S. typhimurium* TA98, without the presence of metabolic activation.

## Introduction

The genus* Artemisia* (Asteraceae tribe Anthemideae) is known to show interesting biological and pharmacological activitie (1, 2). *Artemisia* species are popular plants which are used for the treatment of diseases such as cancer, hepatitis, inflammation and infections by fungi, bacteria, and viruses (3). Within this family, *Artemisia* is included in the tribe of Anthemideae which is comprised of more than 500 species. Among these species, 34 have been reported in Iran, some of which are endemic (4, 5). Among them,* Artemisia ciniformis* Krasch. & Popov ex Poljakov., grows naturally in wide regions of Iran. Previously, the essential oil of *A. ciniformis* has not been characterized with respect to its biological, toxicological and pharmaceutical activities. Due to the presence of compounds with cytotoxic and anti-tumor activities in some plants and significant efficacy of plant derived compounds in inhibition and treatment of cancer, as well as the amazing diversity of natural compounds in plants, the researchers tried to find new phytochemicals and evaluate their biological effects as an essential research priority (6). Therefore, the aims of this study were to study the chemical constituents, antimicrobial, cytotoxicity, mutagenic and anti-mutagenic activities of the essential oil of *A. ciniformis *growing wild in Iran.

## Experimental

*General*

The major equipment types used were a clevenger apparatus, Shimadzu UV-2501PC spectrophotometer (Shimadzu, Japan) and DNM-9602G ELISA reader (Perlong Group, Beijing, China). Microbial and cell culture media and laboratory reagents were from Merck, Germany. The mutagens 2-nitrofluorene, sodium azide and 2-aminoanthracene were also from Merck (Germany). All chemicals were of analytical grade. The human normal healthy lymphocyte and cervical carcinoma HeLa cell lines were obtained from Pasteur Institute, Tehran-Iran.

*Plant material*

The aerial parts of *A. ciniformis* were collected in october 2011 from Baam village, after Gahreman abad in Esfarayen, Province of Khorasan, in northeastern Iran. Voucher specimens have been deposited at the Herbarium of the Research Institute of Forests and Rangelands (TARI), Tehran, Iran. Plant specimen was identified by Dr. Vali-Aallah Mozaffarian from the same institute. The voucher specimen (No. 12569) has been deposited in the herbarium, Department of Pharmacognosy, Faculty of Pharmacy, Mashhad University of Medical Sciences, Mashhad, Iran.

*Isolation of the essential oil*

The leaves of *A. ciniformis *were dried at room temperature for several days. Air-dried leaves of *A. ciniformis *(100 g) were separately subjected to hydrodistillation using a clevenger-type apparatus for 3 h. After decanting and drying the oil over anhydrous sodium sulfate, the oil was recovered. Results showed that essential oil yield was 1.05% (w/w).

*Gas Chromatography*

GC analysis was performed on a Schimadzu 15A gas chromatograph equipped with a split/splitless injector (250 ºC) and a flame ionization detector (250 ºC). Nitrogen was used as a carrier gas at a flow rate of 1 mL/min. The capillary column was DB-5 (50 m × 0.2 mm, film thickness, 0.32 µM). The column temperature was kept at 60 ºC for 3 min, then heated to 220 ºC at a 5 ºC/min rate, and then kept constant at 220 ºC for 5 min. Relative percentages were calculated from peak areas by a Schimadzu C-R4A Chromatopac Data Processor without the use of correction factors.

*Gas Chromatography/Mass Spectroscopy*

GC/MS analysis was performed using a Hewlett-Packard 5973 instrument with an HP-5MS column (30 m × 0.25 mm; film thickness, 0.25 µM). The column temperature was kept at 60 ºC for 3 min, programmed to 220 ºC at a rate of 5 ºC/min, and kept constant at 220 ºC for 5 min. The flow rate of helium as carrier gas was 1 mL/min. The mass spectra were taken at an electron impact energy of 70 eV. The retention indices for all components were determined according to the van den Dool method, using *n*-alkanes as standards. The compounds were identified by comparison of their relative retention indices (RRI, DB5) with those reported in the literature and by comparison of their mass spectra with the Wiley library or with the published mass spectra (7).

*Antimicrobial Activity*

*Oil dilution solvent*

Bacterial strains were streaked on Mueller Hinton agar plates using sterile cotton swabs. Five microlitres of DMSO (dimethylsulphoxide), loaded on sterile blank disks, were placed on the agar plates and were incubated at 37 ºC for 24 h. There was no antibacterial activity on the plates and hence DMSO was selected as a safe diluting agent for the oil. Five microlitres of each oil dilution, followed by sterilization, using a 0.45 µM membrane filter, were added to sterile blank discs. The solvent also served as control.

*Microbial strain and growth media*

*Escherichia coli* (ATCC25922),* Staphylococcus aureus *(ATCC25923),* Pseudomonas aeruginosa *(ATCC8830),* Candida albicans *(ATCC 5027) and *Acinetobacter baumannii* (ATCC 17978) were employed in the study. Nutrient agar was used. Bacterial suspensions were made in brain heart infusion (BHI) broth to a concentration of approximately 10^8^ cfu/mL. Subsequent dilutions were made from the above mentioned suspension, which were then used in the tests.

*Oil sterility test*

In order to ensure sterility of the oil, geometric dilutions of the oil, ranging from 0.036 to 72.0 mg/mL, were prepared in a 96-well microtitre plate, including one growth control (BHI + Tween 80) and one sterility control (BHI + Tween 80 + test oil). Plates were incubated under normal atmospheric conditions at 37 ºC for 24 h. The contaminating bacterial growth, if at all, was indicated by the presence of a white ‘‘pellet’’ on the well bottom.

*Disc diffusion method*

The agar disc diffusion method was employed for the determination of antibacterial activities of the oil in question. 0.1 mL from 10^8^ cfu/mL bacterial suspension was spread on the Mueller Hinton Agar (MHA) plates. Filter paper discs (6 mm in diameter) were impregnated with 5 µL of the undiluted oil and were placed on the inoculated plates. These plates, after remaining at 4 ºC for 2 h, were incubated at 37 ºC for 24 h. The diameters of the inhibition zones were measured in millimeters. All tests were performed in triplicate.

*Determination of minimum inhibitory (MIC) and bactericidal (MBC) concentrations*

All tests were performed in brain heart infusion (BHI) broth supplemented with Tween 80 detergent (final concentration of 0.5% (v/v)). Test strains were suspended in BHI broth to give a final density of 10^7^ cfu/mL and these were confirmed by viable counts. The minimal inhibitory concentration (MIC) and minimal bactericidal concentration (MBC) were assessed according to our modified procedure (8). MIC was determined by a broth dilution method in test tubes as follows: 40 µL from each of various dilutions of the oils were added to 5 mL of brain heart infusion (BHI) both in tubes containing 10^7^ cfu/mL of live bacterial cells. The tubes were then incubated on an incubator shaker to evenly disperse the oil throughout the broth in tubes. The highest dilution (lowest concentration), showing no visible growth, was regarded as the MIC. Cell suspensions (0.1 mL) from the tubes showing no growth were subcultured on BHI agar plates in triplicate to determine if the inhibition was reversible or permanent. MBC was determined as the highest dilution (lowest concentration) at which no growth occurred on the plates.

*Bactericidal kinetics of the oil*

Forty microlitres of each oil at the dilution determined by MBC, was added to each 5 mL of brain heart infusion (BHI) broth in tubes containing bacterial suspension of 10^7^ cfu/mL and were then incubated at 37 ºC in an incubator shaker. Samples (0.1 mL) were taken after 5, 10, 15, 20, 25, 30, 45, 90, 120, 150, 180, 210 and 240 min. The samples were immediately washed with sterile phosphate buffer, pH 7.0, centrifuged at 10000 rpm/1 min, resuspended in the buffer and were then spread-cultured on BHI agar for 24 h at 37 ºC. Phosphate buffer was used as diluent when needed. Bactericidal experiments were performed three times. Microbial colonies were counted from triplicates after the incubation period and the mean total number of viable cells per ml was calculated. The mean total number of viable bacteria from bactericidal kinetics experiments at each time interval was converted to log_10_ viable cells using routine mathematical formulae. The trend of bacterial death was plotted graphically.

*Cytotoxicity assay*

Cytotoxicity was measured using a modified MTT assay on normal human lymphocytes and cancer cells. The human cervical carcinoma HeLa cell lines NCBI code No. 115 (ATCC number CCL-2) were obtained from Pasteur Institute, Tehran-Iran. The cells were grown in RPMI 1640 supplemented with 10% fetal calf serum, 1% (w/v) glutamine, 100 U/mL penicillin and 100 μg/mL streptomycin. The human normal healthy lymphocyte cell lines NCBI code No. 124 (ECACC number 91112124) were obtained from Pasteur Institute, Tehran-Iran. The cells were grown in RPMI 1640 supplemented with 10% FBS. Cells were cultured in a humidified atmosphere at 37 °C in 5% CO_2_. Cytotoxicity assay detected the reduction of MTT [3-(4,5-dimethylthiazolyl)-2,5-diphenyltetrazolium bromide] by mitochondrial dehydrogenase, to the blue formazan product, which reflected the normal functioning of mitochondrial and cell viability (9). Briefly, the cells (5 × 10^4^) were seeded in each well containing 100 μL of the RPMI medium supplemented with 10% FBS in a 96-well plate. After 24 h of adhesion, a serial of doubling dilution of the essential oil was added to triplicate wells over the range of 1.0-0.005 μL/mL. The final concentration of ethanol in the culture medium was maintained at 0.5% (volume/volume) to avoid toxicity of the solvent (10). After 2 days, 10 μL of MTT (5 mg/mL stock solution) was added, and the plates were incubated for an additional 4 h. The medium was discarded, and the formazan blue formed in the cells was dissolved with 100 μL dimethyl sulphoxide (DMSO). The optical density was measured at 490 nm using a microplate ELISA reader. The cell viability curves were calculated from cells incubated in the presence of 0.5% ethanol. Cytotoxicity was expressed as the concentration of drug inhibiting cell growth by 50% (IC_50_). All tests and analyses were run in triplicate, and mean values were recorded (HeLa cells; *y* = 1.2827* x *+ 27.327; R^2^ = 0.9815), (Lymphocyte cells;* y* = 0.006* x *+ 27.976; R^2^ = 0.9787).

*Mutagenicity and anti-mutagenicity activity*

*Preparation of metabolic activation system (S9 Mixture)*

The S9 metabolic activator was prepared just before use by adding: phosphate buffer (0.2 M) 500 µL, deionised water 130 µL, KCl (0.33 M) 100 µL, MgCl_2_ (0.1 M) 80 µL, S9 fraction 100 µL, glucose-6-phosphate (0.1 M), 50 µL and NADP (0.1 M) 40 µL. The mixture was kept on ice during testing. S9 fraction, the liver postmitochondrial supernatant of rats treated with the mixture phenobarbital/β-naphthoflavone (PB/NF) to induce the hepatic microsomal enzymes, was purchased from Moltox (11).

*Toxicity determination*

Bacterial toxicity was determined based on the values ​​in Table 1. For the toxicity test, 12 mL of Nutrient agar and 0.50 mL of metabolic activation (S9) mix or Buffer (Phosphate buffer 0.2M, pH 7.4), 0.01 mL of the test chemical dilution and 0.1 mL overnight culture of the *Salmonella* strain were then added to tubes (Table 1.). The contents of the test tubes were then mixed and poured into the surface of Glucose minimal agar plates. (The Glucose minimal agar, consisting of 1.5% agar, 0.02% MgSO_4_.7H_2_O, 0.2% Citric acid, 1% K_2_HPO_4_, 0.35% NaNH_4_HPO_4_.4H_2_O and 2% Glucose). The plates were inverted and placed in a 37 °C incubator for 48 h. The colonies were then counted and the results were expressed as the number of revertant colonies per plate. Comparisons of bacterial counts on test compound plates with bacterial counts on control plates were used to determine growth inhibition (11).

*Mutagenicity and anti-mutagenicity test*

Mutagenic activity was evaluated by the *Salmonella*/microsome assay, using the *Salmonella* *typhimurium* tester strains TA98 and TA100, with (+S9) and without (−S9) metabolization, using the pre-incubation method (12). It is important that the same number of bacteria be used in the preliminary toxicity assay as well as in the definitive mutagenicity assay (11).

*Salmonella* inoculated cultures 15-18 h prior to performing the experiment. Top agar melt supplemented with 0.05 mM histidine and biotin and maintain at 43 °C to 48 °C. To the 13×100 mm sterile glass tubes maintained at 43 °C, add in the following order with mixing after each addition. Each test should be performed using a single batch of reagents, media and agar (11).

**Figure 1:**
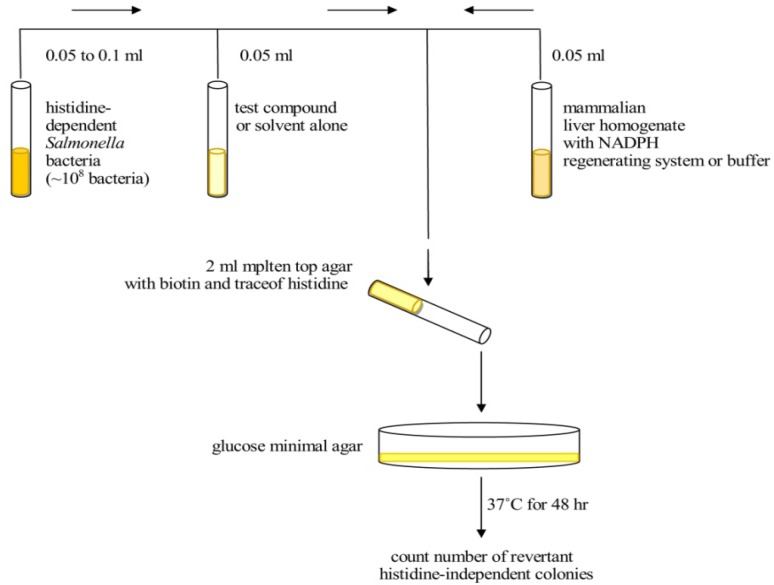
Diagram depicting the steps involved in the plate incorporation assay (9).

The top agar, consisting of 0.6% agar and 0.6% NaCl, is one of the most critical medium components in the Ames test because it contains the trace amount of histidine (0.05 mM) for limited growth. It also contains biotin at a concentration of 0.05 mM which is in excess of what is needed for the growth of the *Salmonella* strains. Using pre-incubation, we studied the effect of metabolic activation. In condition without metabolic activation, 0.01 mL of each concentration of test ingredient, negative control or positive control was added to 0.5 mL of 0.1 M phosphate buffer (pH 7.4) and 0.1 mL of each strain (approximately 1/6×10^6^ cells/mL), and then incubated at 37 ^o^C for 20 min. After shaking incubation, 2 mL of top agar was added to the incubation mixture according to the strains and then poured on to a plate containing minimal agar. The plates were incubated at 37 °C for 48h, and the revertant colonies were counted manually (Figure 1). In the presence of metabolic activation, 0.5 mL of freshly prepared S9 mix instead of 0.1 M phosphate buffer (pH 7.4) were added to the incubation mixture. Other procedures were performed in the same way. All experiments were performed in triplicate (Tables 2-5). The colonies are then counted and the results are expressed as the number of revertant colonies per plate. The standard mutagens used as positive controls in experiments without the S9 mix were 2-nitrofluorene for TA98, sodium azide for TA100. In experiments with S9 activation, 2-aminoanthracene was used with TA98 and TA100. DMSO served as negative (solvent) control (11).

*Results obtained in mutagenicity tests are shown ​​in* Tables 2 *,* 3 *.*

The percentage of mutations was calculated using the following formula:

(T/M)*100

T: The number of revertant colonies in the presence of essential oil

M: The number of revertant colonies in the presence of mutagen

The number of colonies that had been grown up was deducted from the numerator and denominator.

Results obtained in anti-mutagenicity tests were shown ​​in Tables 4,5.

*Statistical analysis*

All analyses and tests were run in triplicate and mean values recorded. All the experimental data are presented as mean ± SEM of three individual samples. IC_50_ value was calculated from the dose-response curves. All of the statistical analyses were performed by means of Microsoft Office Excel 2007 software.

**Figure 2:**
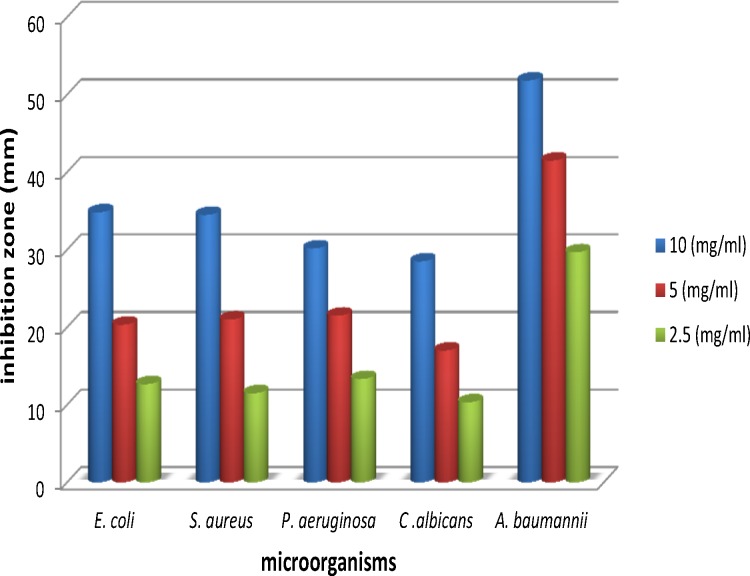
Comparision of antimicrobial activity by disk diffusion method

**Figure 3:**
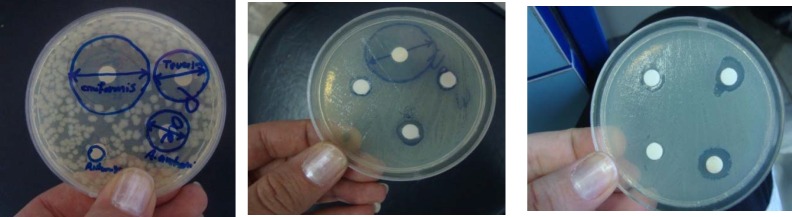
Determination of antimicrobial activity by disk diffusion method

**Figure 4:**
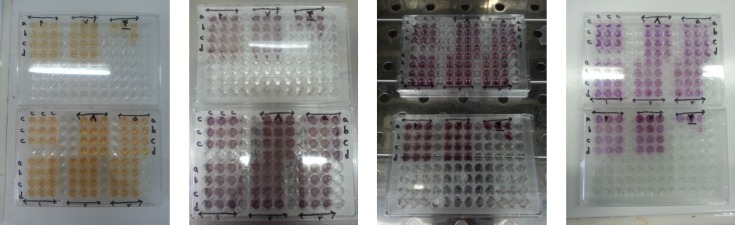
Cytotoxicity tests on Hela and Lymphocyte cells

**Figure 5:**
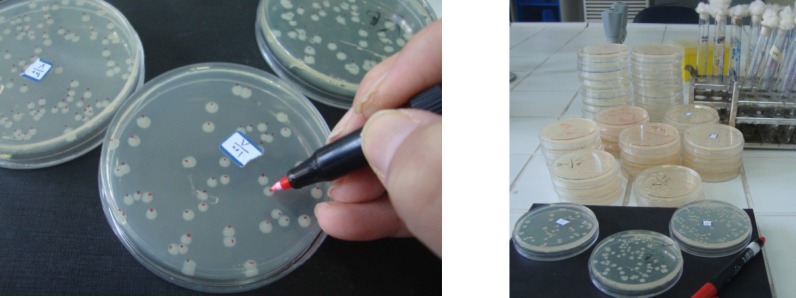
Colonies counting in the Ames test

**Table 1:** Determination of toxicity of the leaf essential oil of *Artemisia ciniformis* Krasch. & Popov ex Poljakov

**Control 2**	**Test2**	**Control** **1**	**Test 1**	**TA100/TA98**
0.1 mL	0.1 mL	0.1 mL	0.1 mL	Test strain (approximately 1/6×10^6^ cells/ml)
-	0.01 mL	-	0.01 mL	Oil
0.6 mL	0.5 mL	0.1 mL	-	Phosphate buffer (0.2M, pH7.4)
-	-	0.5 mL	0.5 mL	S9 mix
12 mL	12 mL	12 mL	12 mL	Nutrient agar

**Table 2:** Determination of mutagenic potency of* A. ciniformis *oil by *S. typhimurium* strains TA100 and TA98 without S9

**Test**	**Negative Control**	**Positive Control**	**TA100/TA98**
0.1 mL	0.1 mL	0.1 mL	Test strain (approximately 1/6×10^6^ cells/mL)
-	-	0.1 mL	sodium azide (NaN_3_) (50 µg/mL) for TA100
			2-nitrofluorene (1.5 µg/plate) for TA98
0.01 mL	-	-	Test concentration (oil)
0.5 mL	0.5 mL	0.5 mL	Phosphate buffer (0.1 M, pH7.4)
2 mL	2 mL	2 mL	Top agar

**Table 3:** Determination of mutagenic potency of* A. ciniformis *oil by *S. typhimurium* strains TA100 and TA98 with S9.

**Test**	**Negative Control**	**Positive Control**	**TA100/TA98**
0.1 mL	0.1 mL	0.1 mL	Test strain (approximately 1/6×10^6^ cells/mL)
-	-	0.1 mL	2-aminoanthracene (1µg/plate in DMSO)
0.01 mL	-	-	Test concentration (oil)
0.5 mL	0.5 mL	0.5 mL	S9 mix
2 mL	2 mL	2 mL	Top agar

**Table 4:** Determination of antimutagenic potency of* A. ciniformis *oil by *S. typhimurium* strains TA100 and TA98 without S9

**Test**	**Negative Control**	**Positive Control**	**TA100/TA98**
0.1 mL	0.1 mL	0.1 mL	Test strain (approximately 1/6×10^6^ cells/mL)
200 µL	-	200 µL	sodium azide (NaN_3_) (50 µg/µL) for TA100
			2-nitrofluorene (1 µg/µL) for TA98
10 µL	-	-	Test concentration (oil)
0.5mL	0.5 mL	0.5 mL	Phosphate buffer (0.1 M, pH7.4)
2 mL	2 mL	2 mL	Top agar

**Table 5:** Determination of antimutagenic potency of* A. ciniformis *oil by *S. typhimurium* strains TA100 and TA98 with S9.

**Test**	**Negative Control**	**Positive Control**	**TA100/TA98**
0.1 mL	0.1 mL	0.1 mL	Test strain (approximately 1/6×10^6^ cells/ml)
200 µL	-	200 µL	2-aminoanthracene (1µg/µl in DMSO)
10 µL	-	-	Test concentration (oil)
0.5 mL	0.5 mL	0.5 mL	S9 mix
2 mL	2 mL	2mL	Top agar

**Table 6:** Chemical constituents of the leaf oil of *A. ciniformis* Krasch. & Popov ex Poljakov

**Percentage(%)**	**RI**	**Compound**	
23.7	1033	1,8-Cineole	
2.46	1068	*cis*-Sabinene hydrate	
12.28	1139	*trans*-Pinocarveol	
30.21	1143	Camphor	
4.86	1162	Pinocarvone	
2.94	1165	Borneol	
3.82	1177	Terpinen-4-ol	
2.45	1189	α**-**Terpineol	
2.67	1194	Myrtenol	
3.21	1217	*trans*-Carveol	
1.76	1298	Carvacrol	
2.15	1404	*z*-Caryophylene	
1.04	1436	Cedrane	
1.23	1439	α**-**Guaiene	
-		Monoterpene hydrocarbons	
90.36		Oxygenated monoterpenes	
4.42		Sesquiterpene hydrocarbons	
-		Oxygenated sesquiterpenes	
-		Others	
94.78		Total	
^*^RI, Retention indices were as determined on a DB-5 column using the homologous series of *n*-alkanes		

**Table 7:** Antimicrobial activity of the essential oil of *A. ciniformis* Krasch. & Popov ex Poljakov

**Microorganisms**			*Escherichia coli* ATCC25922	*Staphylococcus aureus* ATCC25923	*Pseudomonas aeruginosa* ATCC8830	*Candida albicans* ATCC 5027	*Acinetobacter baumannii* ATCC 17978
IZ* (mm)	Oil (mg/mL)	**10**	34.83±1.65	34.50±1.32	30.17±0.76	28.50±1.00	51.83±1.76
		**5**	20.33±2.02	21.00±1.00	21.50±1.50	17.00±0.50	41.50±0.87
		**2.5**	12.67±1.15	11.50±1.32	13.33±0.58	10.33±1.76	29.67±1.53
**MIC** * **-MBC** * **(mg/mL)**			1-2.5	2.5-5	1-2.5	1-2.5	0.02-0.04
**D value** * **(min)**			6.43	17.14	8.57	8.57	3.57

IZ*: Inhibition Zone (mm); MIC*: Minimum Inhibitory Concenteration (mg/mL); MBC*: Minimum Bactericidal Concentration (mg/mL); D-value*: Decimal Reduction Time (minutes

**Table 8:** Cytotoxicity assay of the essential oil of *A. ciniformis *on Hela and lymphocyte cells

**Oil Dilutions (** **µ** **g/mL)**	**% Viable Hela ** **cells**	**% Hela** ** cells** ** Death**
control	100	0
7	78.66 ±5.6	21.33
14	59.34 ±4.75	40.65
28	33.12 ±5.89	66.87
IC_50 _(µg/mL)	19.64	
**Oil Dilutions (** **µ** **g/mL)**	**% Viable Lymphocyte ** **cells**	**% Lymphocyte ** **cells** ** Death**
control	100	0
700	79.45 ±2.31	20.54
1400	71.76 ±7.11	28.23
2800	63.91 ±9.18	36.08
5600	51.71 ±9.18	48.28
IC_50 _(µg/mL)	5711.66	

**Table 9:** Percent of mutagenicity (M) and antimutagenicity (A) of *A. ciniformis* oil to *S. typhimurium* (TA98, TA100) with and without S9

	**Dilution** **(mg/plate)**	**Percent of mutagenicity (M)**			
A. ciniformis oil	0.8	TA 100 M-S9	TA 100 M+S9	TA 98 M-S9	TA 98 M+S9
		85.71	7.50	30.43	5.71

		Percent of antimutagenicity (A)			
		TA 100 A-S9	TA 100 A+S9	TA 98 A-S9	TA 98 A+S9
		13.46	45.83	86.36	58.62

## Results

*Chemical composition of the essential oil*

As it is shown in Table 6, 14 components, representing 94.78% of the total composition, were identified in the leaf oil of *A. ciniformis*. The leaf oil of *A. ciniformis* consists of eleven oxygenated monoterpenes (90.36%) and three sesquiterpene hydrocarbons (4.42%). Camphor (30.21%), 1,8-cineole (23.7%) and *trans*-pinocarveol (12.28%) were the major components in this oil.

*Antimicrobial assays*

Antibacterial and antifungal activities of the oil of* A. ciniformis *were tested by three methods: 1. agar diffusion method, 2. determination of minimum inhibitory (MIC) and 3. minimum bactericidal concentrations (MBC) and bactericidal kinetics of the oil, using different dilutions viz., 2.5 mg/mL, 5 mg/mL and 10 mg/mL. The results of antibacterial activities of the essential oil of *A. ciniformis *are presented in Table 7. Maximum inhibition was obtained against* Acinetobacter baumannii *(51.83 mm),* Escherichia coli *(34.83 mm) and* Staphylococcus aureus *(34.50 mm) followed by* Pseudomonas aeruginosa *(30.17 mm) and* Candida albicans* (28.50 mm) at a concentration of 10 mg/mL of the oil (Figure 2, 3.). The oil of *A. ciniformis *indicated moderate inhibitory activity against all tested microorganisms*. *Complete death time on exposure to *A. ciniformis *oil was 3.57 min for *A. baumannii*. The results showed that *A. baumannii* has the minimum MIC and MBC values against *A. ciniformis *oil.

*Cytotoxicity assay*

Cytotoxicity was measured using a modified MTT assay (Figure 4.) (13). The cytotoxic effects of *A. ciniformis *oil were tested using HeLa and lymphocyte cells (Table 8.). At a concentration of 28 μg/mL, oil inactivated HeLa cells by 66.87%, at lower doses, the oil was tolerated by the cells and its 50% cytotoxic concentration was 19.64 μg/mL. The oil displayed an excellent cytotoxic action towards the human tumor cell line. On the other hand, at a concentration of 5600 µg/mL, oil inactivated lymphocytes by 48.28%. Thus, the oil exerted a highly significant cytotoxic effect on the human tumor cell line.

*Mutagenicity and anti-mutagenicity test*

The Ames assay is commonly used to detect mutagenic and antimutagens activities and is a widely accepted method for identifying different chemicals and drugs that can cause gene mutations. In this study, the concentration of the oil (0.8 mg/plate) was selected based on a preliminary toxicity test. The colonies were counted to determine the mutagenic and antimutagenic potencies of *A. ciniformis *oil (Figure 5.). Mutagenesis and antimutagenesis percentages of *A. ciniformis *oil against* S.* *typhimurium* strains TA98 and TA100, with (+S9) and without (−S9) metabolization were calculated and listed in Table 9.

The maximum percentage of anti**-**mutagenicity of *A. ciniformis *oil was seen in 0.8 mg/plate against the 2-nitrofluorene by the bacterial reverse mutation assay in the strain of* S. typhimurium* TA98, without the presence of metabolic activation S9.

## Discussion

*Chemical composition of the essential oil*

Oxygenated monoterpenes especially camphor, 1,8-cineole and *trans*-pinocarveol were the major constituents in the leaf oil of *A. ciniformis* collected from Esfarayen in Iran, in october 2011. In contrast, the aerial parts of *A. ciniformis *Krasch. & M. Pop. ex Poljak, which were collected from the Bojnourd area of Iran in November 2005, consisted of ten monoterpene hydrocarbons (35.5%), seven oxygenated monoterpenes (23.1%), and four sesquiterpenes (33.9%). Davanone (29.6%), myrcene (14.4%), camphor (10.6%), p-cymene (9.6%) and linalool (8.6%) were found to be the major components among the 22 constituents characterized, comprising 92.5% of the total components detected (14).

*Antimicrobial assays*

In the present study, determination of MBC and MIC from the oil of *A. ciniformis *indicated that all the test organisms were approximately sensitive to the oil, but *A. baumannii* was the most vulnerable one. In this study, bactericidal kinetics of the oil of *A. ciniformis *indicated that *A. baumannii* is the most vulnerable. To the best of my knowledge, there has been no research about the antimicrobial effect of the essential oil of *A. ciniformis*.

*Cytotoxicity assay*

The IC_50_ shows that cytotoxicity of the oil towards human tumor cell line is much higher than that required for human healthy cells. These results indicate low adverse side effects of the oil. The tested oil seemed to deserve further investigation. Although all *in-vitro* experiments hold limitations with regards to possible *in-vivo* efficacy, the results of this study are very promising with regards to possible antineoplastic chemotherapy and form a very sound basis for future research. Essential oil components have a very different mode of action in eukaryotic and bacterial cells. For bacterial cells, they are having strong bactericidal properties, while in eukaryotes, they modify apoptosis and differentiation, interfere with the post-translational modification of cellular proteins, induce or inhibit some hepatic detoxifying enzymes. So, essential oils may induce very different effects in prokaryotes and eukaryotes (15).

In previous investigation it has been shown that the extract of *A. ciniformis *leads to a decrease in the reactive oxygen species (ROS) generation. The obtained results showed that only ethyl acetate, ethanol and ethanol/water extracts of *A. ciniformis* are able to protect H9c2 cardiomyoblast cells against H_2_O_2_ cytotoxicity (16).

*Mutagenicity and anti-mutagenicity test*

The essential oil of *A. ciniformis *showed excellent antimutagenicity effect in the presence of 2-nitrofluorene, in the strain of* S. typhimurium* TA98, without the presence of metabolic activation. On the other hand, the mutagenic activity of *A. ciniformis *oil in the strain of* S. typhimurium* TA100, without the presence of metabolic activation S9 was more than its antimutagenic activity against sodium azide. But, in the presence of chemical mutagens, the oil of *A. ciniformis *showed resistance against this mutagens and has anti mutagenic effects. The large scale use of essential oils requires accumulation of toxicological data on these substances. There is a relationship between mutagenesis and carcinogenesis.

 The Ames mutagenicity assay is a short**-**term bacterial reverse mutation assay specifically designed to detect a wide range of chemicals which produces genetic damage leading to gene mutations. These mutations are the consequence of DNA damage induced by different mechanisms. 

To our knowledge, there was no research carried out on the mutagenic and anti-mutagenic effect of essential oil of *A. ciniformis *before. In the present study, the anti**-**mutagenic effect of *A. ciniformis *oil was observed at 0.8 mg/plate in the bacterial reverse mutation assay in both strains of* S. typhimurium* TA100 and TA98.

## Conclusion

The results obtained from human cells and *Salmonella typhimurium* suggest that the essential oil of *A. ciniformis *may be exploited as a natural anti HeLa cells, antimicrobial source and anti-mutagenic agent with low adverse side effects. The results of this study are very promising with regard to possible anti**-**neoplastic chemotherapy and form a basis for future studies.
